# The Meninges as CNS Interfaces and the Roles of Meningeal Macrophages

**DOI:** 10.3390/biom15040497

**Published:** 2025-03-28

**Authors:** Chihiro Hiraki, Fuminori Tsuruta

**Affiliations:** 1 Master’s and Doctoral Program in Biology, Degree Programs in Life and Earth Sciences, Graduate School of Science and Technology, University of Tsukuba, 1-1-1 Tennodai, Tsukuba 305-8577, Ibaraki, Japan; s2320951@u.tsukuba.ac.jp; 2 Master’s and Doctoral Program in Biology, Institute of Life and Environmental Sciences, University of Tsukuba, 1-1-1 Tennodai, Tsukuba 305-8577, Ibaraki, Japan; 3 Doctoral Program in Human Biology, Graduate School of Comprehensive Human Sciences, University of Tsukuba, 1-1-1 Tennodai, Tsukuba 305-8577, Ibaraki, Japan; 4 Doctoral Program in Humanics, School of Integrative and Global Majors, University of Tsukuba, 1-1-1 Tennodai, Tsukuba 305-8577, Ibaraki, Japan; 5 Master’s and Doctoral Program in Neuroscience, Graduate School of Comprehensive Human Sciences, University of Tsukuba, 1-1-1 Tennodai, Tsukuba 305-8577, Ibaraki, Japan; 6 Center for Quantum and Information Life Sciences, University of Tsukuba, 1-1-1 Tennodai, Tsukuba 305-8577, Ibaraki, Japan

**Keywords:** CNS-associated macrophages, microglia, meninges, brain vessels, CNS interface

## Abstract

The brain, the most important component of the central nervous system (CNS), is protected by multiple intricate barriers that strictly regulate the entry of proteins and cells. Thus, the brain is often described as an organ with immune privilege. Within the brain parenchyma, microglia are thought to be the primary resident immune cells, with no other immune-related cells present under normal conditions. On the other hand, recent studies in the meningeal border regions have revealed the presence of meningeal-specific lymphatic vessels and channels that connect to the skull bone marrow. Importantly, resident macrophage populations specific to these boundary regions, known as CNS-associated macrophages (CAMs) or border-associated macrophages (BAMs), have been identified. In contrast to the brain parenchyma, the meninges contain many immune-related structures and cells, making them an important immune interface at the CNS border. CAMs serve a dual function, triggering immune responses under pathological conditions and supporting the maintenance of brain homeostasis. This review focuses on the immune architecture of the meninges and the roles of CAMs in humans and mice, summarizing and discussing recent advances in this field.

## 1. Introduction

The brain, as the main part of the central nervous system (CNS), comprises the parenchyma, which is responsible for essential brain functions such as regulating learning and memory, emotions, motor control, and bodily homeostasis. Furthermore, CNS interfaces, such as meninges and brain vessels, exhibit structural characteristics distinct from those of the parenchyma and play important roles in regulating such brain functions.

Traditionally, immune cells in the CNS were thought to consist solely of microglia within the parenchyma [1]. However, resident macrophages with phenotypes distinct from microglia, termed CNS-associated macrophages (CAMs), have been rediscovered in the CNS interfaces [2,3,4,5,6,7]. CAMs are subdivided into several types depending on their location, particularly in the meninges situated between the skull and the cerebral cortex, where they exhibit further phenotypic specialization [8]. Moreover, recent studies in the meninges have identified novel mechanisms for immune cell trafficking, including lymphatic vessels previously thought absent from brain regions and channels connecting to skull bone marrow. These findings highlight a new role for the meninges as an interface between the nervous and immune systems within brain regions [9,10,11,12]. In this review, we describe a comprehensive summary of recent research on CAMs and other immune components related to the meninges and immune systems in mice and humans.

## 2. Structure and Function of the Meninges

**Figure 1:**
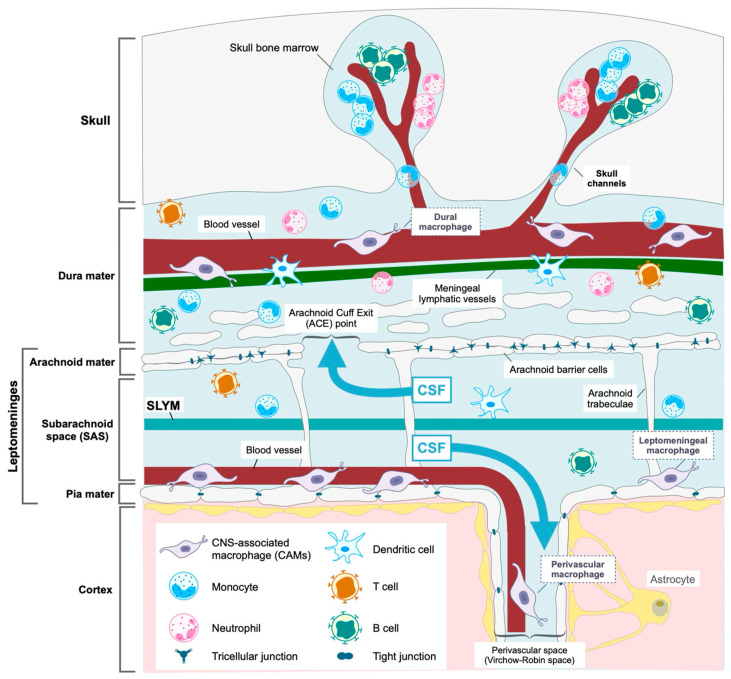
The structure of the meninges.

The meninges are structures located between the cerebral cortex and the skull, composed of multiple layers, each with distinct structural and functional characteristics [13]. Previously, three membranous layers have been identified in the meninges: the dura mater, the arachnoid mater, and the pia mater. More recently, a fourth layer, the subarachnoid lymphatic-like membrane (SLYM), which lies between the arachnoid and pia mater, has been reported [14]. From a functional perspective, the meninges are commonly classified into two compartments: the “dura” compartment and the “leptomeninges” compartment, which includes the arachnoid and pia mater (Figure 1). This section will explore the unique characteristics of each meningeal layer and discuss the functional roles of these two compartments.

The meninges are a layered membranous structure comprising the dura mater, arachnoid mater, and pia mater, along with the recently identified SLYM. The leptomeninges and dura mater are strictly compartmentalized. The subarachnoid space exists between the arachnoid mater and pia mater. Blood vessels traverse the subarachnoid space, and CSF circulates in it. The dura mater contains blood vessels, dural lymphatic vessels, and channels connecting to the cranial bone marrow. Unlike the cortex, these regions host diverse immune cell pools, including CAMs, which function as resident macrophages in the meninges.

### 2.1. The Leptomeninges

The leptomeninges comprises the pia mater and the arachnoid mater. The pia mater is the innermost layer of the meninges [13]. It adheres closely to the cerebral cortex and consists of a single cell layer tightly bound by tight junctions, along with a basement membrane known as the pial basement membrane. This close association with the cerebral cortex and the glial limitans formed by astrocyte end-feet provides an effective separation for the cortex and contributes to the brain’s immune privilege [15]. Blood vessels extending from the leptomeninges into the cerebral cortex form a perivascular space created as the blood-brain barrier develops around them. The pia mater is continuous, with the basement membrane surrounding these perivascular spaces [16].

In contrast to the pia mater, the arachnoid mater forms the boundary with the more apical dura mater. The arachnoid mater is composed of several layers of flattened cells connected by adherens junctions [13]. Additionally, a layer of arachnoid barrier cells is present between the arachnoid and dura mater, where these cells establish a boundary through tight junctions. Recent studies have shown that the fibroblasts forming these layers are subdivided into clusters according to their specific layers, with varying adhesion mechanisms [16,17]. In fibroblasts that constitute the arachnoid layer, cellular retinoic acid-binding protein 2 (*Crabp2*) is expressed in arachnoid cells but absent in pia mater cells, indicating that proteins with distinct expression profiles can mark the different layers [16]. Previously, arachnoid barrier cells were believed to form a boundary solely through tight junctions. However, recent studies present an alternative perspective. Transcriptomic analysis of fibroblasts forming the arachnoid barrier cells has revealed the expression of not only tight junction-associated proteins such as *Cldn11* and *Tjp1* (tight junction protein 1, also known as zonula occludes 1, *ZO-1*) but also tricellular junction proteins, including *angulin-1* (also known as *Lsr*, or lipolysis-stimulated lipoprotein receptor) and *angulin-3* (also known as *Ildr2*, or immunoglobulin-like domain containing receptor 2). Further observation using electron microscopy has shown that arachnoid barrier cells form a bilayered cellular structure featuring complex attachments through tight junctions, adherens junctions, and tricellular junctions [16].

The leptomeninges is completely isolated by these membranes, creating a space between the two layers known as the subarachnoid space (SAS). The SAS is supported by arachnoid trabeculae, structures composed of pial cells and collagen fibers [18], and is traversed by blood vessels and filled with cerebrospinal fluid (CSF). Endothelial cells lining the blood vessels in the SAS are joined by tight junctions, forming a barrier that completely separates blood from CSF [19,20].

One of the most critical functions of the leptomeninges is its role as a circulation pathway for CSF. CSF functions as the interstitial fluid of CNS, filling the meninges and ventricles [21]. Unlike peripheral tissues, the brain parenchyma and leptomeninges lack lymphatic vessels. Therefore, a mechanism distinct from the traditional lymphatic system is required for the clearance of waste products and tissue fluid circulation within the brain. The recently proposed glymphatic system is considered a novel lymphatic-like mechanism in the brain [22,23,24]. In this system, CSF in the SAS infiltrates the cerebral cortex through the perivascular spaces and is similarly cleared via these spaces. As mentioned earlier, the pia mater is continuous with the basement membrane of the perivascular spaces around vessels entering from the meninges connecting the SAS with the perivascular spaces [16]. This structure allows CSF in the SAS to flow into the arterial perivascular spaces. Due to the high pulsatile pressure exerted by the heartbeat on arteries, CSF is pushed toward the brain parenchyma through aquaporin-4 channels expressed in the end-feet of astrocytes that form the blood-brain barrier, where it mixes with the interstitial fluid filling the parenchyma [22]. Subsequently, old interstitial fluid containing waste products effluxes back into the SAS through the venous perivascular spaces. This flow enables the circulation of CSF within the SAS, facilitating clearance within the cerebral cortex.

Arachnoid barrier cells in the arachnoid mater, forming the boundary with the dura mater side of the SAS [25], are partially discontinuous, creating “Arachnoid Cuff Exit” (ACE) points through which CSF is drained to the dura mater [26]. These points provide a direct connection between the SAS and the dura mater. CSF flows through ACE points into the dura mater, eventually reaching the cervical lymph nodes through lymphatic vessels in the dura mater, and is subsequently drained into the peripheral lymphatic system [9,10]. In this way, the SAS facilitates the inflow and outflow pathways for circulating CSF at the surface of the cerebral cortex. Since the outflowing CSF has cleared the cerebral cortex, it is expected to contain various solutes, including waste products, in contrast to the CSF before it enters circulation. However, the precise mechanisms by which the inflowing and outflowing CSF maintain their distinct pathways remain unclear.

One potential explanation for this issue is the presence of the fourth meningeal layer, the “SLYM” [14,27]. The SLYM was discovered through in vivo two-photon excitation microscopy in *Prox1-EGFP^+^* reporter mice, which marks Prospero homeobox protein 1 (*Prox1*), a transcription factor involved in lymphatic fate determination. The SLYM is a monolayer membrane of *Prox1-EGFP^+^* cells containing collagen fibers, positioned to bisect the SAS horizontally. Claudin-11 (*CLDN-11*), expressed in arachnoid barrier cells, is absent in *Prox1-EGFP^+^* SLYM cells. Moreover, the SLYM exhibits a distinct expression profile characterized by lymphatic-like features (*Prox1-EGFP^+^*, *PDPN^+^*, *LYVE1^−^*, *CRABP2^+^*, *VEGFR3^−^*, *CLDN-11^−^*, and *E-Cad^−^*), which do not align with the cell types of the dura mater, arachnoid mater, or pia mater.

Podoplanin (*PDPN*), a lymphatic marker distinct from *Prox1*, is known to be expressed in mesothelial cells that line body cavities [28]. Mesothelial cells are thought to act as a lubricant on boundaries, enabling the smooth movement of tissues. Accordingly, the SLYM might serve a similar function in reducing friction between the brain and the skull during cranial movement. Additionally, one of the SLYM’s key features is its role in restricting molecular movement [14]. When tracers are injected into either the outer SAS (above the SLYM) or the inner SAS (below the SLYM), these molecules remain separate, with no mixing observed. This molecular restriction also applies to small dextran molecules as small as 3 kDa, suggesting that the SLYM could limit the movement of fine solutes within the CSF. Although the precise CSF flow routes remain unclear, this compartmentalization in the SAS implies the possibility that distinct CSF pathways are maintained before and after entering the cerebral cortex.

However, the existence of the SLYM has also been questioned [16,27]. Typically, detachment of the skull is necessary when sampling brain tissue, during which the SLYM is believed to be torn. As a result, a membrane corresponding to the SLYM is not usually observed in standard brain sections. If the SLYM does indeed exist, this characteristic might explain why its discovery was delayed compared to the other three meningeal layers.

### 2.2. The Dura Mater

The dura mater is a dense collagen layer that attaches to the inner side of the skull [13]. It is divided into two layers. The outer layer functions as the periosteum on the inner surface of the skull, while the inner layer is mostly adhered to the outer layer but separates in areas near the venous sinuses. The dura mater folds to form structures known as dural reflections, which include the falx cerebri, separating the cerebral hemispheres, and the tentorium cerebelli, which separates the cerebrum from the cerebellum [29]. The dura contains numerous blood vessels, unlike the blood vessels in the leptomeninges, as it lacks tight junctions that form barriers [30]. As a result, the dura allows for more active movement of molecules and cells from the blood, in contrast to the leptomeninges and the cerebral cortex. The most notable feature of the dura is its abundance of immune-related structures. The term “immune privilege” has long been used to describe the brain and its surrounding regions, which were thought to be deficient in immune structures and lymphatic tissues [31]. However, numerous immune structures have recently been discovered in the dura mater alone, prompting a surge in research on its role as an immune hub for the brain. Here, we will focus on the dural lymphatic vessels and associated tissues [9,10], as well as the channels connecting the skull bone marrow and dura mater [11,32,33].

The dural lymphatic vessels were initially suggested in anatomical research as early as the 18th century [34], but their existence was not widely accepted until they were rediscovered in 2015 [9,10]. Unlike typical lymphatic vessels, the network of dural lymphatic vessels has low coverage of the brain and markedly lower complexity [9]. At the top of the skull, these vessels are distributed near the eyes, adjacent to the paranasal sinuses, passing over the olfactory bulb and along the dural sinuses. It has been observed that dural lymphatic vessels around the transverse sinus are thicker and more branched than those near the superior sagittal sinus. Additionally, these vessels run along the skull base and at the junction between the skull and neck, ultimately connecting to the deep cervical lymph nodes.

Tracer studies have shown that substances injected into the brain parenchyma or ventricles are cleared along these dural lymphatic vessels toward the deep cervical lymph nodes. In contrast, the absence of dural lymphatic vessels results in reduced clearance of large molecules and their decreased drainage to the deep cervical lymph nodes, indicating that these vessels play a critical role in the clearance of large molecules from the brain [10].

Dural lymphatic vessels have few valves to prevent backflow of lymphatic fluid [9]. Near the dural sinuses at the top of the skull, valves are almost nonexistent, with only a few found in the relatively large lymphatic vessels at the skull base. These lymphatic vessels have been identified as afferent lymphatic vessels. They express classic lymphatic endothelial markers such as *Lyve1* and *Prox1* at the molecular level. Additionally, punctate expression signals of *Claudin-5* and vascular endothelial (VE) cadherin have been observed, suggesting characteristics similar to initial lymphatic vessels. However, due to the scarcity of valves, there is no expression of integrin α9, a marker characteristic of lymphatic valves. While dural lymphatic vessels lack smooth muscle cells—a unique feature—they largely resemble peripheral lymphatic vessels in structure.

Dural lymphatic vessels play a vital role in the drainage of interstitial fluid from the brain and the transport of immune cells. These vessels connect to the deep cervical lymph nodes, allowing CSF from the surrounding environment to be transported to peripheral lymph nodes via the deep cervical lymph nodes. Experiments involving the injection of tracers into CSF and the subsequent destruction of dural lymphatic vessels demonstrated a lack of tracer spread across brain regions near blood vessels. This suggests that dural lymphatic vessels contribute to the diffusion of CSF. In addition to CSF, dural lymphatic vessels transport proteins contained within the CSF [35]. For instance, in Alzheimer’s disease, damage to dural lymphatic vessels is known to lead to the accumulation of amyloid-β (Aβ) in the meninges and accelerate its deposition in the cerebral cortex [35,36].

Furthermore, dural lymphatic vessels contain immune cells, including T cells, B cells, and bone marrow-derived cells expressing MHC class II, responsible for transporting immune cells to brain regions [37,38,39,40]. Recent findings have revealed that, along with blood vessels within the dura mater, these immune cell structures form what is referred to as dural associated lymphoid tissue (DALT) [41]. This lymphoid structure, located near the blood vessels within the dura mater, forms a complex structure near the rostral nasal confluence of the sinuses, referred to as a lymphatic hub. This hub comprises various immune cells, such as CD11c^+^ bone marrow-derived cells, CD3^+^ T cells, and B cells, along with PDPN^+^ fibroblastic reticular cells and LYVE1^+^ lymphatic vessels. When fluorescent tracers are injected into veins, they leak out along venous sinuses and are taken up by B cells and macrophages within DALT. Furthermore, introducing lipopolysaccharide (LPS) into veins leads to the proliferation of CD45^+^ cells and B cells within this hub, indicating that circulating antigens can activate immune cells in DALT. Additionally, upon viral infection through the nasal passages, a significant increase in immune cell clusters, such as CXCR5^+^ B cells and CD45^−^ CXCL13-expressing cells, has been observed. This response reveals that these lymphoid structures also play a role in local immune responses surrounding the CNS. DALT exists in a steady state and is thought to provide a rapid immune response to pathogens entering from the nasal passages, thereby protecting brain regions.

Another distinctive structure in the cranial area is the channel connecting the skull bone marrow to the dura mater [11,33,42,43]. These channels, known as “skull channels”, are found in the inner cortex of the skull and are surrounded by osteoblasts on the bone surface [11], essentially forming holes within the skull itself. CD31, a marker of endothelial cells, is observed within these channels, indicating the presence of vascular structures. These channels are distributed throughout the skullcaps, with a higher density in the frontal and occipital regions [32]. Notably, the channels in the occipital region are slightly longer and wider in diameter than those in the frontal and parietal regions, showing structural heterogeneity as reported in studies using X-ray computed tomography analysis. Inside the channels, CD31 expression signals the presence of blood vessels surrounded by a certain amount of open space. This space enables not only the passage of blood vessels but also facilitates CSF transport [11]. Experiments involving tracer injections into the cisterna magna of mice have shown that the tracers appear near the blood vessels within the skull bone marrow, suggesting a pathway where CSF flows from the subarachnoid space to the dura mater and then through the channels into the skull bone marrow [12,32].

In addition to CSF, these channels also allow for the transport of cells [11,32,38,40,42]. Under normal conditions, CSF flows from the dura mater into the skull bone marrow cavity. However, in cases of bacterial meningitis or stroke, bone marrow-derived cells migrate against this flow direction, moving toward the dura mater [11,32]. During such brain invasions or inflammation within the brain, an increase in myeloid cells within the skull bone marrow has been observed, and these cells have been shown to migrate to the meninges [12,32]. This suggests that the skull channels not only serve as a conduit for fluids but also play a crucial role in immune responses by enabling the movement of immune cells in response to CNS inflammation or infection.

## 3. Macrophages in the Meninges

The meninges play an important role in maintaining the homeostasis of the cerebral cortex, where the blood-brain barrier restricts the movement of molecules and cells. Accordingly, the meninges contains various types of immune cells [2,3,4,8,37,44]. This paragraph will focus on immune cells within the meninges, with particular attention to a kind of cells that have recently garnered interest: CAMs.

### 3.1. Development of CAMs

**Figure 2:**
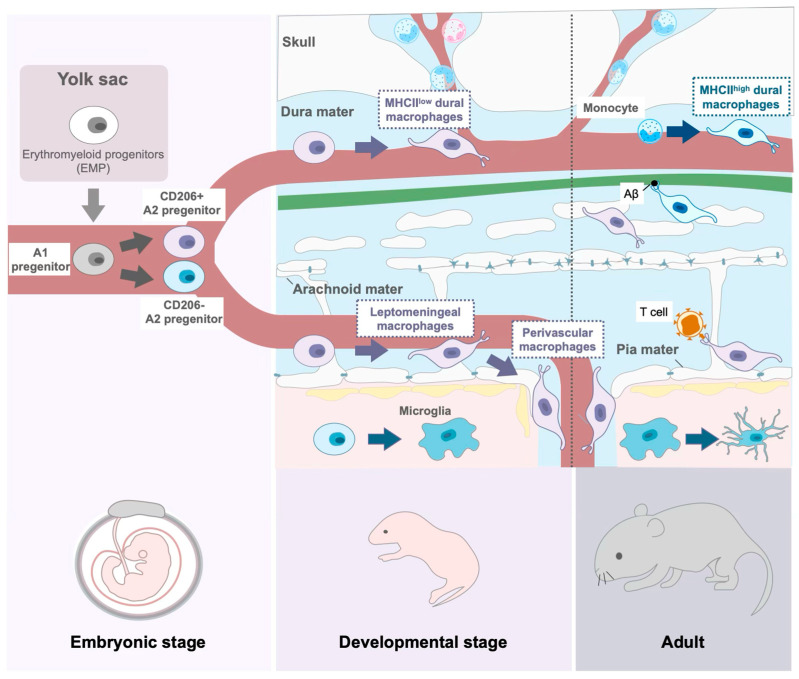
The overview of CAM development.

Macrophages, among immune cells, are long-lived cells of the innate immune system. They play a key role in recognizing pathogens, engaging in phagocytosis and presenting antigens to T cells to initiate the adaptive immune response [45,46,47]. Since macrophages develop in nearly all tissues during ontogeny, each tissue hosts specific resident macrophages with unique functions. The brain is no exception, containing two primary types of resident macrophages: microglia and CAMs (Figure 2) [2,3,4,44]. Microglia are the only immune cells residing in the brain parenchyma. They dynamically alter their characters depending on developmental stages and microenvironmental changes, supporting the acquisition and maintenance of proper brain function [1,48,49,50,51,52]. While most hematopoietic cells circulating in the blood originate from hematopoietic stem cells (HSCs) [53], resident macrophages such as microglia and CAMs have a distinct developmental origin. They derive from primitive macrophages formed from erythromyeloid progenitors (EMP) in the yolk sac during the embryonic stage [44,54]. EMPs are present in the yolk sac as early as embryonic day (E) 8.5 and reach the brain by E9.5 via the developing fetal circulation [55]. At this stage, CAMs are exclusively observed in the meninges; they are absent in perivascular spaces and the choroid plexus, suggesting that the meninges are the first brain region reached by CAMs [44]. Although microglia also arrive in the brain around E9.5, their exact route and mechanism of infiltration into the parenchyma remain unclear.

During embryonic development, CAMs infiltrate the brain via fetal circulation and differentiate into resident macrophages. Based on CD206 expression, CD206^−^ cells develop into microglia, whereas CD206^+^ cells differentiate into CAMs. The leptomeningeal macrophages serve as a source of perivascular macrophages during development. In adult mice, microglia exhibit increased heterogeneity, and dural macrophages are replaced by monocyte-derived cells, whereas leptomeningeal macrophages retain their EMP origin. In the postnatal meninges, CAMs play key roles in waste clearance and frontline immune defense against pathogens.

By E10.5, two distinct populations of primitive macrophages have been identified in both the yolk sac and the brain [56]. One group expresses signature CAM genes like *Lyve1*, *Ms4a4a*, and *CD206*, while the other exhibits a high expression of signature genes characteristic of adult microglia, including *Sall1*, *Hexb*, and *P2ry12*. These populations are believed to correspond to adult CAMs and microglia, respectively, and both maintain their unique phenotypes from early development through to E18.5. The phenotypes observed during early development correspond to the gene expression profiles of mature CAMs and microglia, indicating an early onset of these cell identities. Interestingly, both CAMs and microglia express *Tgfbr2*, a receptor for TGF-β signaling, which plays a critical role in microglial differentiation. When *Tgfbr2* is deleted, there is a decrease in microglial population throughout development, and the remaining microglia exhibit reduced expression of microglial signature genes while upregulating CAM markers like *TIM-4*, *CD204*, and *CD206*. In contrast, CAMs exhibit no changes in cell number or phenotype, demonstrating that while TGF-β is essential for microglial differentiation, it does not influence CAM phenotype regulation. Thus, the early differentiation of microglia and CAMs originates from two distinct macrophage populations in the yolk sac and brain and is further defined by TGF-β signaling within the brain [56].

Recent fate-mapping studies have identified CD206^+^ microglial progenitors during early embryonic days 10.5 to 14.5 [57]. Initially thought to be CAMs located in perivascular spaces, these CD206^+^ cells were later found to infiltrate the cortex as microglial-like cells, preceding CAM migration into the perivascular spaces [57]. Additionally, time-lapse imaging experiments using brain slices have revealed that CD206^+^ cells infiltrate the cortex and gradually acquire microglia-like characteristics [58]. These CD206^+^ cells, considered to be CAMs arriving in the brain during early development, cross the rooftop of the developing brain around embryonic day 12.5 to enter the ventricular space. Following this, CD206^+^ macrophages migrate from the ventricle into the cortex, where they reside for a certain period, during which they lose their CAM-like characteristics and acquire traits like microglia. These findings suggest that microglia and CAMs are not solely determined by intrinsic programs of the two primitive macrophage lineages from the yolk sac and early brain but can differentiate according to specific brain regions.

Recent research has shown that, during early postnatal development, the propagation of neuronal micronuclei to microglia changes their characteristics [59]. Microglia incorporating micronuclei undergo morphological changes, such as a decrease in process complexity. Interestingly, microglia incorporating micronuclei exhibit an increased expression of signature genes associated with CAMs. Thus, microglia and CAMs may acquire their specific traits through intrinsic programming and region-dependent niche signals that direct their differentiation based on their localization, despite sharing a common origin.

On the other hand, while CAMs exhibit a homogeneous phenotype during early development, they acquire distinct regional characteristics in the adult stage [2,3,8]. For instance, while microglia typically exhibit a highly branched morphology with dynamic cell processes and a stable cell body, CAMs located in the leptomeninges (leptomeningeal macrophages) display an amoeboid shape with a cell body that is itself highly motile [44]. In contrast, CAMs in the perivascular space (perivascular macrophages) do not move their cell bodies but are capable of extending and retracting processes along the perivascular area [4,44]. This extension and retraction activity is known to increase during brain inflammation [4]. This adaptive morphology and motility in CAMs, which varies according to their regions, highlights functional distinctions from microglia despite their shared origin. However, because these region-specific CAMs share known cell markers, identifying each type of CAM has traditionally been challenging without considering its anatomical context.

With advancements in single-cell RNA sequencing (scRNA-seq) technologies, the classification of immune cells within the brain has become increasingly detailed [8]. In scRNA-seq analyses of the brain’s boundary regions, CAMs, which were previously broadly categorized into four subsets based on their localization —perivascular macrophages, choroid plexus macrophages, dural macrophages, and leptomeningeal macrophages—they have now been further subdivided into six distinct subsets. This refined classification highlights the heterogeneity and functional diversity of CAMs. This section will focus on the detailed characteristics of meningeal macrophages.

Leptomeningeal macrophages are classified as a single subset in this study, whereas dural macrophages are divided into two subsets based on their expression levels of MHC II proteins. Both subsets share common transcriptional signatures, including genes such as *Apoe*, *Ms4a7*, *Ms4a6c*, *Lyz2*, and *Tgfbi*. However, each subset also exhibits unique expression profiles. For instance, CD206 shows differential expression between the two subsets of dural macrophages.

Numerous differentially expressed genes have also been identified between the MHC II-high and MHC II-low macrophage populations in the dura, highlighting their heterogeneity even within the same anatomical region. In the MHC II-high subset, the progressive expression of monocyte-associated genes, such as *Ccr2*, suggests that a portion of dural macrophages may originate from monocytes. At birth [postnatal day (P) 0], nearly all CAMs exhibit high CD206 expression and lack MHC II expression. However, starting around P21, dural macrophages undergo significant phenotypic changes, including the downregulation of CD206. By 20 weeks postnatally, the CD206-low, MHC II-high macrophage population becomes the dominant subset, representing a dramatic shift in cellular composition.

Further evidence from lineage-tracing experiments using mice and capable of labeling bone marrow-derived macrophages reveals stark differences in the origins of CAMs between regions. Microglia and leptomeningeal macrophages retain their yolk sac-derived populations throughout life. In contrast, dural macrophages, which are initially yolk sac-derived at early developmental stages, are gradually replaced by bone marrow-derived macrophages over time. This replacement underscores a striking distinction between CAMs in neighboring regions: leptomeningeal macrophages maintain their embryonic origins, while dural macrophages are supplanted by secondary hematopoietic monocyte-derived populations. These developmental origin differences are believed to contribute to the regional diversity of CAMs in adulthood. Furthermore, the selective replacement of CAMs in the dura—but not in the leptomeninges—may be attributed to the dural environment’s lack of a blood-CSF barrier. This open access to the peripheral immune system likely facilitates the dynamic cell turnover observed in the dura, contrasting with the more insulated environment of the leptomeninges. While significant progress has been made in understanding the cellular development of CAMs, the regulation of their protein-level development and the mechanisms driving their phenotype acquisition remain largely unexplored. Recent scRNA-seq analyses of developmental-stage CAMs have identified several transcription factors that may be master regulators of CAM development [8,44].

*Spic* and *Irf7* are believed to play a universally critical role across all subsets of CAMs [8]. Beyond these shared master regulators, it has been suggested that each subset possesses distinct transcriptional regulators that drive its unique phenotype(s) [8,44]. For instance, in the leptomeninges macrophage and MHC II-low dural macrophage population, transcription factors such as *Maf* and *Etv1* have been identified as key regulators [8]. Conversely, in the MHC II-high dural macrophage cluster, which is predominantly composed of monocyte-derived cells, transcription factors including *Runx3* and *Bcl3* are thought to be significant. These findings underscore the presence of both shared and subset-specific transcriptional programs underlying CAM development and functional specialization. Key transcription factors regulating microglial development include *Sall1*, *Mafb*, and *Irf8* [8,44,60], highlighting the fact that CAMs are governed by transcriptional regulatory mechanisms distinct from those that govern microglia. This also underscores the unique phenotype acquisition mechanisms associated with the developmental origins of each subset of CAMs.

Interestingly, the absence of *Irf8*, a transcription factor critical for microglial phenotype regulation, affects not only microglia but also leptomeninges macrophages [8,44,60]. While the core signatures of CAMs remain unchanged in *Irf8*-deficient cells, numerous differentially expressed genes have been identified. Many of these genes are associated with immune responses, with gene ontology analyses indicating enrichment in categories such as innate immune response and defense response to other organisms [56]. This suggests that CAMs functions are transcriptionally regulated by *Irf8*. Notably, while *Irf8* deficiency causes phenotypic changes in microglia, such as morphological alterations, CAMs exhibit no such changes beyond gene expression differences. Furthermore, the disruption of other key factors involved in microglial phenotype regulation, such as *Mafb* or the downstream signaling molecule *Smad4* in the TGF-β pathway, significantly impacts microglial characteristics but has minimal effects on CAMs. These findings highlight the differential regulatory mechanisms between microglia and CAMs [61,62].

Despite these advances, the functional relevance of candidate regulatory factors for CAMs in vivo remains largely uninvestigated. Future research is anticipated to provide deeper insights into the transcriptional control and developmental mechanisms underpinning CAMs specialization and functionality. One of the important roles of leptomeninges macrophages during development is to serve as a source of perivascular macrophages. These macrophages reside within the perivascular space, a specialized compartment surrounding cortical blood vessels, which are shielded by the blood-brain barrier [2,4,63]. This barrier is formed by astrocytic end-feet and vascular endothelial cells, among other components [20]. The perivascular space gradually expands starting around P5, coinciding with the emergence of perivascular fibroblasts and the progressive maturation of the blood-brain barrier [57]. In response to this physical niche expansion, leptomeningeal macrophages migrate into the perivascular space, where they differentiate into perivascular macrophages. Notably, perivascular macrophages are absent immediately after birth and begin to appear around P10 [44,57]. Their population continues to increase until approximately two weeks after birth, after which it stabilizes [57]. This expansion in cell number depends on both sustained infiltration from the meninges and local proliferation within the perivascular space. The occupation of the perivascular niche by leptomeningeal macrophages is mediated by integrin signaling. Deletion of *Tln1* (Talin-1), a key regulator of integrin-mediated signaling, results in a specific reduction in the number of perivascular macrophages while leaving microglia and meningeal macrophages unaffected.

### 3.2. Function of CAMs in Meninges

In the homeostatic brain, CAMs are exposed to the flow of CSF, which facilitates the immune surveillance of CSF and the exchange of antigens, metabolites, and other molecules. Regarding the meninges, studies have reported reduced antigen drainage from the CNS parenchyma and subsequent accumulation of dural macrophages in response to the obstruction of dural lymphatic vessels [35]. However, whether dural macrophages actively participate in antigen drainage remains unclear. On the other hand, research involving direct tracer injection into the CSF via the cisterna magna has demonstrated that leptomeningeal macrophages and perivascular macrophages internalize some tracers [64]. Furthermore, depletion of CAMs in the leptomeninges and perivascular spaces using clodronate liposomes resulted in decreased CSF influx and efflux, suggesting a potential role for these CAMs in regulating CSF dynamics. In CAMs-depleted mice, the diameter of the perivascular space was reduced, and extracellular matrix (ECM) proteins such as type IV collagen and laminin accumulated within the perivascular region. Macrophages are generally known to regulate ECM remodeling through matrix metalloproteinases (MMPs) production [45,65]. In CAMs-depleted mice, decreased MMPs activity was observed, supporting the hypothesis that CAMs influence ECM composition in the perivascular space [64]. Taken together, these findings suggest that leptomeningeal and perivascular CAMs regulate CSF flow by modulating ECM remodeling within the perivascular niche.

In the meninges, dural macrophages function as the first responders to microbial invasion in the brain [66]. They release cytokines such as interferon-beta (IFN-β), interferon-gamma (IFN-γ), and tumor necrosis factor (TNF) to recruit peripheral immune cells. A study involving the depletion of dural macrophages followed by lymphocytic choriomeningitis virus infection revealed an impaired immune response [32]. Mice lacking both meningeal macrophages and T cells exhibited increased viral load in the brain and higher mortality rates, highlighting the critical role of meningeal macrophages in antigen presentation and immune defense.

An association between meningeal CAMs and Alzheimer’s disease (AD) has also been suggested. AD is characterized by neuronal death and localized microgliosis due to aggregates like Aβ plaques and tau neurofibrillary tangles [35,67]. Proteins linked to AD, such as clusterin (CLU), apolipoprotein E (APOE), and amyloid precursor protein (APP) are believed to be cleared from the cortex via the glymphatic system. Depletion of leptomeningeal and perivascular macrophages has been shown to result in their accumulation in CSF [22,68,69]. Moreover, the absence of dura mater lymphatic vessels exacerbates the accumulation of these proteins [64]. However, the direct relationship between their clearance and meningeal macrophage remains to be fully elucidated.

The role of CAMs in CNS disorders, including multiple sclerosis and Parkinson’s disease, as well as cerebrovascular diseases, has been extensively studied, primarily focusing on perivascular macrophages [2,3,4,70]. However, reports directly linking meningeal CAMs to these conditions are scarce. Meningeal imaging poses challenges due to its location under the skull, as live imaging often involves invasive procedures like skull thinning, which can disrupt meningeal integrity. Additionally, distinguishing CAMs from peripheral bone marrow-derived cells recruited during injury or disease complicates the study of meningeal CAM functions.

## 4. Conclusions

The meninges, situated at the interface of the brain and its immune privilege, represent a unique and dynamic area of research. Recent discoveries highlight the meninges’ structural and functional importance, especially in maintaining CNS homeostasis and immune responses. Key roles such as those played by meningeal lymphatic vessels and the glymphatic system emphasize their function beyond being a physical barrier. Meningeal CAMs, identified via scRNA-seq, display diverse origins and regional variability, which is crucial for CNS development. However, understanding their broader physiological roles remains a significant challenge, promising insights into CNS immunity and homeostasis.

## Data Availability

Not applicable.
